# Predicting and Monitoring Upper-Limb Rehabilitation Outcomes Using Clinical and Wearable Sensor Data in Brain Injury Survivors

**DOI:** 10.1109/TBME.2020.3027853

**Published:** 2021-05-21

**Authors:** Sunghoon I. Lee, Catherine P. Adans-Dester, Anne T. O’Brien, Gloria P. Vergara-Diaz, Randie Black-Schaffer, Ross Zafonte, Jennifer G. Dy, Paolo Bonato

**Affiliations:** Department of Physical Medicine and Rehabilitation, Harvard Medical School, and also with the College of Information and Computer Sciences, University of Massachusetts Amherst.; Department of Physical Medicine and Rehabilitation, Harvard Medical School.; Electrical and Computer Engineering, Northeastern University.; Department of Physical Medicine and Rehabilitation, Harvard Medical School, at Spaulding Rehabilitation Hospital, Boston, MA 02115 USA; Wyss Institute for Biologically Inspired Engineering, Harvard University, Cambridge, MA 02138 USA

**Keywords:** Gaussian Process Regression, Machine Learning, Precision Rehabilitation, Stroke, Traumatic Brain Injury, Wearable Technology

## Abstract

**Objective:**

Rehabilitation specialists have shown considerable interest for the development of models, based on clinical data, to predict the response to rehabilitation interventions in stroke and traumatic brain injury survivors. However, accurate predictions are difficult to obtain due to the variability in patients’ response to rehabilitation interventions. This study aimed to investigate the use of wearable technology in combination with clinical data to predict and monitor the recovery process and assess the responsiveness to treatment on an individual basis.

**Methods:**

Gaussian Process Regression-based algorithms were developed to estimate rehabilitation outcomes (i.e., Functional Ability Scale scores) using either clinical or wearable sensor data or a combination of the two.

**Results:**

The algorithm based on clinical data predicted rehabilitation outcomes with a Pearson’s correlation of 0.79 compared to actual clinical scores provided by clinicians but failed to model the variability in responsiveness to the intervention observed across individuals. In contrast, the algorithm based on wearable sensor data generated rehabilitation outcome estimates with a Pearson’s correlation of 0.91 and modeled the individual responses to rehabilitation more accurately. Furthermore, we developed a novel approach to combine estimates derived from the clinical data and the sensor data using a constrained linear model. This approach resulted in a Pearson’s correlation of 0.94 between estimated and clinician-provided scores.

**Conclusion:**

This algorithm could enable the design of patient-specific interventions based on predictions of rehabilitation outcomes relying on clinical and wearable sensor data. *Significance:* This is important in the context of developing precision rehabilitation interventions.

## I. Introduction

STROKE and traumatic brain injury (TBI) are major causes of long-term disability [1], [2]. They often result in significant motor impairments with negative impact on patients’ independence and quality of life [3], [4]. The recovery of motor abilities in these patients is marked by two phases: in the subacute phase, patients typically display a spontaneous biological recovery [5], [6]; in the chronic phase, they show limited and rather unpredictable motor gains in response to rehabilitation [7], [8]. These observations have been interpreted by some as evidence that traditional rehabilitation interventions have a modest impact on the recovery trajectory, and thus, new intervention approaches should be explored [9]. Consequently, researchers have made many efforts toward investigating new approaches based, for instance, on delivering higher dosage interventions than typically done in the clinic. Studies have reported mixed results with some suggesting that an increase in therapy dosage does not necessarily lead to improvements in motor abilities [10], while others suggesting that significant motor gains can be achieved with high-dosage interventions enabled by technologies such as robotics and functional electrical stimulation [11]. Researchers have also investigated the use of approaches designed to enhance the effects of rehabilitation interventions by facilitating plasticity and motor learning (e.g., transcranial direct current stimulation), with mixed results in both stroke and TBI survivors [12], [13]. Furthermore, approaches combining pharmacotherapies and motor training have been a topic of investigation because of their potential for enabling motor gains in patients who would be otherwise unresponsive to rehabilitation interventions [14], [15]. However, studies combining pharmacotherapies and motor training have also reported mixed results [16]–[18].

Whereas the availability of many rehabilitation approaches provides the opportunity to design patient-specific interventions and thus to achieve optimal clinical outcomes, the criteria that should be used to select the most suitable intervention given the individual patient’s characteristics are still debated. Among others, Winstein and Varghese proposed investigating mechanistic approaches to the design of rehabilitation interventions [19]. These approaches could rely on new computational models of plasticity and learning [20], as well as new data analysis techniques designed to unravel the mechanisms underlying the generation of patterns of motion and muscular activity [21], [22], thus shedding light onto the effects of patients’ injuries on the generation of motor outputs and enabling the design of patient-specific interventions. The hope is that these techniques would allow clinicians to identify and target patient-specific abnormalities in the generation of motor outputs and hence obtain large motor gains in patients who are not responsive to traditional rehabilitation interventions.

Ideally, the design of rehabilitation interventions would rely on predicting and longitudinally monitoring clinical outcomes, which in turn would allow clinicians to select the intervention associated with optimal outcomes on a patient-by-patient basis. In this context, clinicians and researchers have directed their attention to the use of wearable sensors as a tool to assess the effects of rehabilitation interventions [23], [24]. In some of these studies, researchers developed techniques to monitor stroke survivors’ upper-limb motor performance in the home and community setting using accelerometers placed bilaterally on the wrists or fingers [25]–[28]. A variety of measures quantifying the amount of stroke-affected limb use compared to the contralateral limb have been employed, such as the ratio of limb use intensity and the ratio of limb use duration, which were computed based on counts of activity level. However, these methods do not capture the quality of upper-limb movement performance, which is an important aspect of rehabilitation outcomes [29]. In other studies, researchers focused their efforts on estimating clinical outcome measures using inertial data captured during the performance of motor tasks associated with clinical assessments. This body of work includes methods to estimate Functional Ability Scale (FAS) scores derived during the performance of tasks of the Wolf Motor Function Test (WMFT) [30], Fugl-Meyer Assessment Upper Extremity (FMA-UE) scale scores [31]–[33] and metrics of arm use highly correlated with Box and Block Test scores [34]. Unfortunately, these studies have been limited to the analysis of cross-sectional data. This body of work has not addressed yet the need for predicting and monitoring longitudinal changes in motor abilities and thus assess if individual patients are responsive to the prescribed therapeutic intervention.

Using data collected by means of wearable sensors to estimate the motor recovery trajectory of stroke and TBI survivors is meaningful only if the technology provides researchers and clinicians with predictions of rehabilitation outcomes that are more accurate than the ones clinicians can derive based on information that is routinely available in the clinic (e.g., motor impairments and functional limitations at baseline, age of the subject, dosage of the intervention, etc.). It is worth emphasizing that clinical evaluations are regularly performed prior to starting rehabilitation interventions. Hence, information about the patients’ status and the intervention characteristics are known to the clinical team. Would this clinical information be sufficient to accurately predict rehabilitation outcomes in response to the intervention? Would it be necessary to use wearable sensor data to obtain accurate predictions of the rehabilitation outcomes? Would combining clinical data-based estimates and wearable sensor data-based estimates lead to improvements in the accuracy of the predictions of rehabilitation outcomes?

**Fig. 1.**
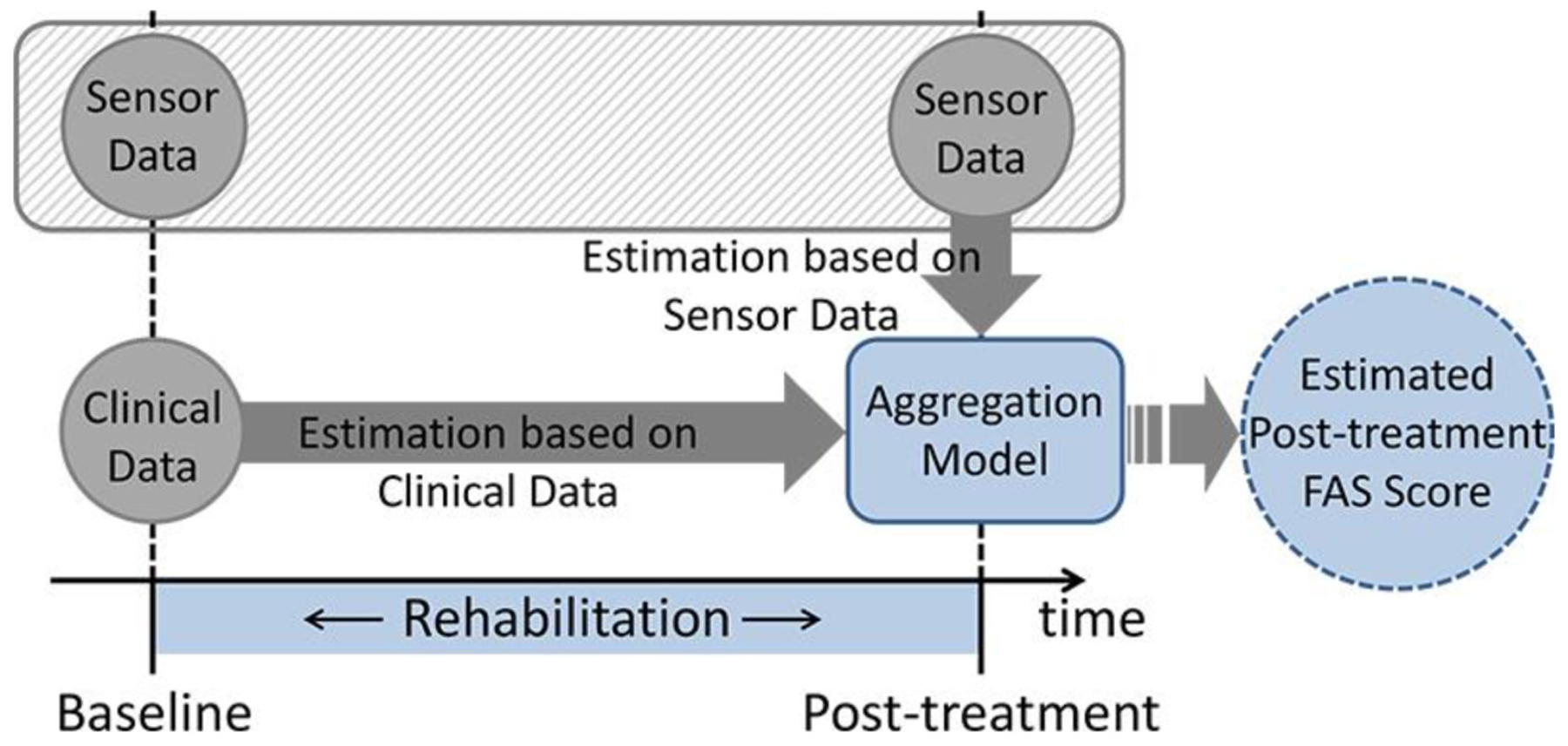
Schematic representation of the approach to predicting and monitoring rehabilitation outcomes investigated in the study. Functional Ability Scale (FAS) scores are estimated independently using sensor data and using clinical data. Then these estimates are combined using an aggregation model based on machine learning.

In this study, we addressed the above-stated questions by collecting longitudinal data from a cohort of stroke and TBI survivors with a broad spectrum of baseline clinical characteristics and by developing algorithms (Fig. 1) suitable to generate estimates of FAS clinical scores, a clinical scale used to assess the quality of patients’ upper-limb movements. Specifically, we developed two distinct Gaussian Process Regression (GPR) models using as input 1) data collected using wearable sensors and 2) data routinely available in the clinic. Then, we introduced and evaluated a constrained linear model that combines the two estimates derived using sensor data and clinical data, respectively. A comparison of the results obtained using these different algorithms allowed us to identify a suitable approach to predict and longitudinally monitor rehabilitation outcomes in stroke and TBI survivors.

## II. Materials and Methods

### A. Subjects and Data Collection Procedures

A total of 44 subjects with upper-limb hemiparesis were enrolled in the study. Of these subjects, 22 were stroke survivors (53.7 ± 17.2 years old - mean ± standard deviation -, 1.06 ± 1.47 years post stroke), and 22 were TBI survivors (33.1 ± 16.0 years old, 0.35 ± 0.62 years post injury). Subjects were considered eligible to participate in the study if they were 1) 18–80 years old, 2) beginning to undergo an inpatient or outpatient rehabilitation intervention involving upper-limb therapy at the time of the baseline visit, and 3) affected by upper-limb impairments as determined using the FMA-UE scale (i.e., score between 15 and 55 of the 66 points of the scale). All the subjects participated in a first study visit and 38 of them also participated in a second study visit after undergoing a rehabilitation intervention. Six subjects were unable to return to the laboratory for the second visit. Of the subjects who participated in the second study visit, 17 were stroke survivors (55.2 ± 16.1 years old, 0.97 ± 1.51 years post stroke) and 21 were TBI survivors (32.4 ± 15.9 years old, 0.39 ± 0.64 years post injury). Eight of the 17 stroke survivors and 20 of the 21 TBI survivors who completed both study visits were rehabilitation inpatients at the start of the study. All the other subjects were outpatients.

**Fig. 2.**
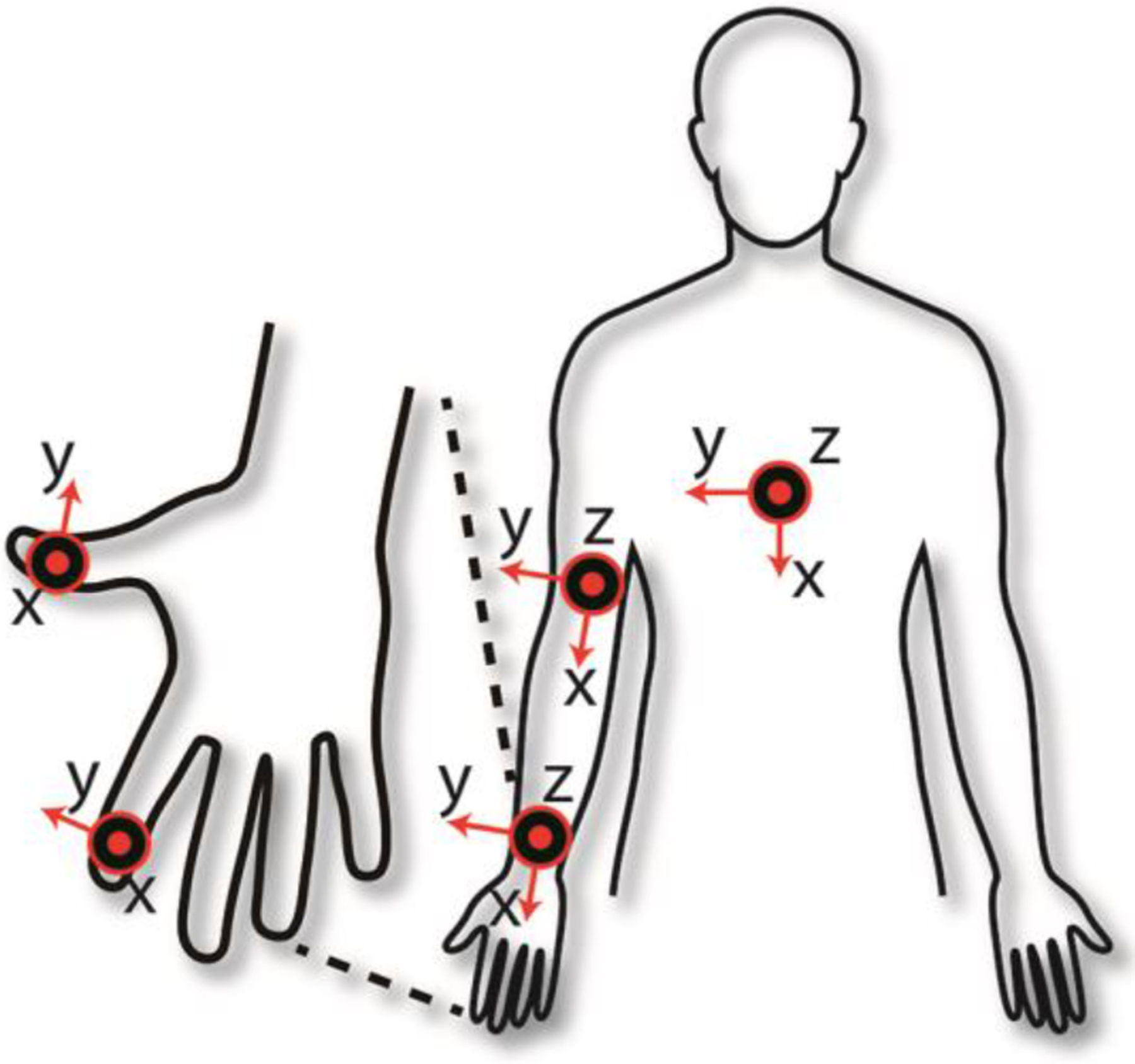
Placement and orientation of the sensors utilized to collect accelerometer data. The sensors placed on the sternum, arm, and wrist were three-axis accelerometers, whereas those on the fingers were two-axis accelerometers.

During the study visits, wearable accelerometer units (Shimmer2 units by Shimmer Sensing, Dublin, Ireland) were secured using bi-adhesive tape and/or Velcro straps to the sternum as well as the arm, wrist, thumb, and index finger of the hemiparetic upper-limb. Fig. 2 shows a schematic representation of the placement of the sensor units. The units secured to the sternum, arm, and wrist were equipped with three-axis accelerometers. The units secured to the thumb and index finger were equipped with two-axis accelerometers. It is noteworthy that we did not use gyroscopes or magnetometers in this study based on prior research that showed satisfactory estimation performance for motor ability in stroke survivors solely using accelerometer data [30], [35]. Each sensor streamed accelerometer data to a base station using dedicated software that provided time synchronization of the sensor units.

After donning the sensor units, subjects were instructed to perform a series of motor tasks to assess the quality of upper-limb movements using the FAS [30]. The FAS is a clinical scale that relies on visual inspection of the patterns of motion during the performance of the WMFT [36]–[40]. The quality of the patterns of motion is assessed using a six-point ordinal scale (0–5). A score of 0 is used when the subject is unable to or does not attempt to perform the task. A score of 5 is used when the subject completes the task by displaying patterns of movement marked by characteristics that are indistinguishable from those displayed by subjects with no motor impairments. Scores from 1 to 4 are used to capture incremental levels of severity deviating from normative motor behaviors, with focus on factors such as speed, coordination, accuracy, effort, smoothness, and the presence of synergy and compensatory movements in completing the task.

**Fig. 3.**
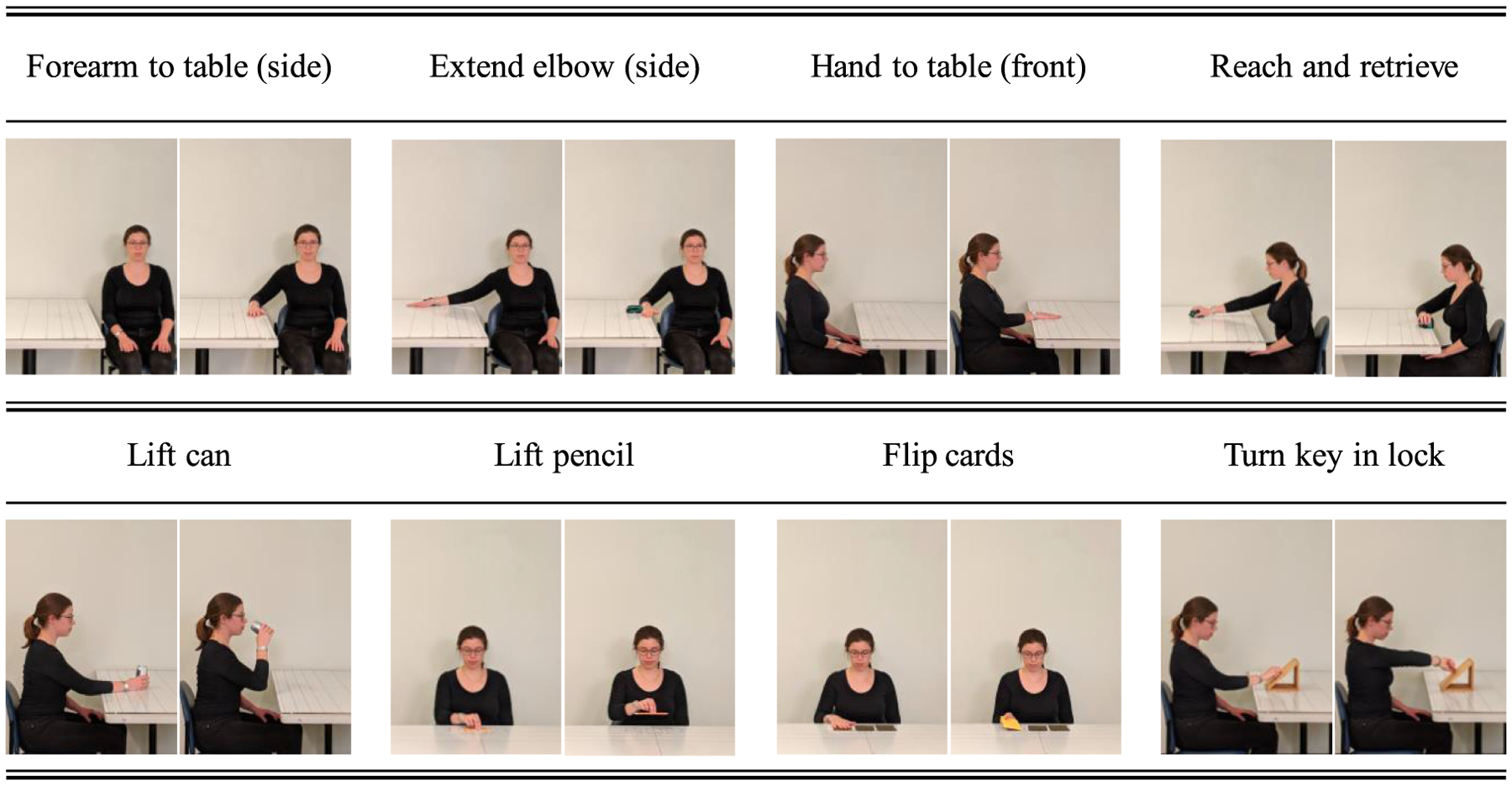
A research staff member demonstrating the motor tasks from the Wolf Motor Function test (WMFT) that were used to derive Functional Ability Scale (FAS) scores. The first four tasks (top row) are herein referred to as *Reaching Tasks*. The other four tasks (bottom row) are referred to as *Manipulation Tasks*.

In the study, subjects performed eight of the 15 motor tasks pertaining to the WMFT, which were previously shown to be suitable to allow clinicians to accurately estimate the total FAS score [30]. Fig. 3 shows the eight motor tasks that were performed by the study participants using the stroke/TBI-affected limb: 1) moving the forearm from the lap to a table positioned on the affected-limb side of the subject - referred to as “forearm to table (side)”, 2) starting with the arm on a table, extending the elbow against a weight - extend elbow (side), 3) moving the hand from the lap to a table positioned in front of the subject - hand to table (front), 4) pulling a weight toward the body - reach and retrieve, 5) reaching for a soda can on a table, bringing it close to the mouth (i.e. pretending to drink from the soda can), and positioning it back on the table - lift can, 6) picking up a pencil using the thumb and first two fingers - lift pencil, 7) flipping three cards positioned on a table - flip cards, and 8) reaching for a key in a lock and turning it - turn key in lock. The first four of these tasks are herein referred to as *Reaching Tasks*, whereas the remaining tasks are referred to as *Manipulation Tasks* [30].

Subjects were instructed to perform each of the above-listed motor tasks three times during each study visit, consistently with a prior study by our research group [30]. The sessions were video-recorded and later reviewed by three trained clinicians. Clinicians were asked to provide FAS scores for a randomly selected but approximately equal number of video recordings. In the randomization process, data collected during each subject’s study visit were considered as a single block, namely the data was assigned to a single clinician. FAS scores were provided for each repetition of each motor task. Because the clinicians received the same training to administer the FAS and because previous studies showed high within- and between-rater reliability for the FAS, with an Intra-class Correlation Coefficient (ICC) value between 0.88 and 0.95 [38], [41] and a weighted *ĸ* value of 0.8 [42], we did not deem as necessary to ask multiple clinicians to provide FAS scores for the same recordings or to assure that the same clinician generated FAS scores for baseline and post-treatment recordings from a given subject. However, we tracked the variability in the FAS scores for the three repetitions of each motor task performed by each subject. Specifically, we estimated the average value of the standard deviation of the FAS scores for each subject and each task. We observed a variability of approximately 2.5%, which is negligible from a clinical point of view.

All the study procedures were approved by the Spaulding Rehabilitation Hospital Institutional Review Board (IRB). All study participants signed an IRB-approved informed consent form describing the experimental protocol and the health risks and benefits associated with the study.

### B. Overview of the Data Analysis Pipeline

**Fig. 4.**
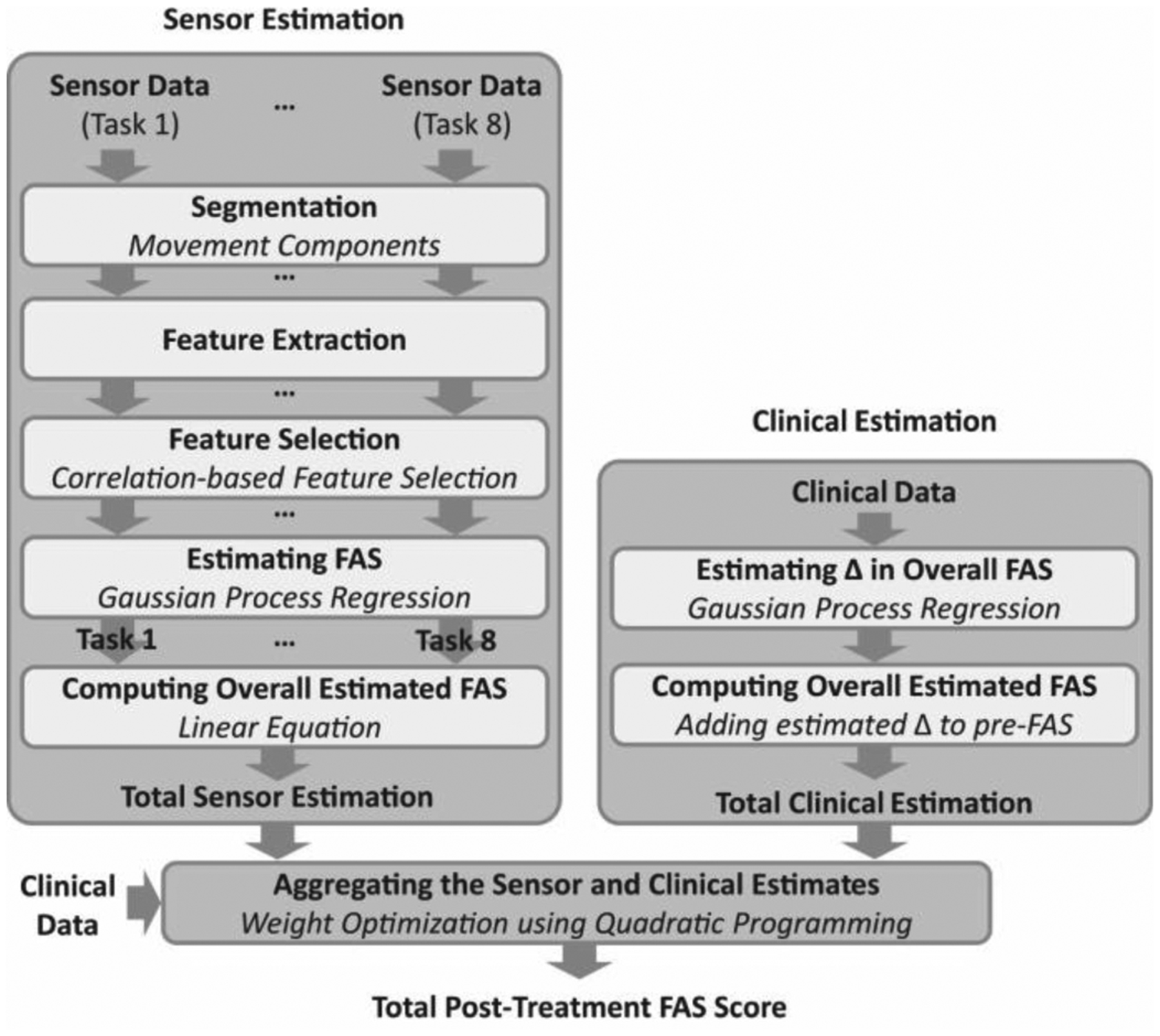
Graphical illustration of the data analysis pipeline developed in the study to derive rehabilitation outcome estimates (i.e., FAS scores) using the sensor data (block diagram on the left), the clinical data (block diagram on the right), and by combining these estimates using a machine learning model (block diagram on the bottom).

Fig. 4 shows a schematic representation of the developed data analysis pipeline, which included separate algorithms to generate FAS score estimates using solely the sensor data (block diagram on the left), using solely the clinical data (block diagram on the right), as well as combining these two estimates using weights derived by means of an optimization technique that relied on clinical data (block diagram on the bottom).

### C. Sensor Data-Based Estimation

In this section, we provide a description of the algorithm to derive sensor data-based estimates. The algorithm consisted of a cascade of processing modules to 1) automatically segment the accelerometer time-series, 2) extract data features, 3) select data features suitable to accurately estimate the FAS scores, and 4) generate FAS score estimates. The latter module consisted of a GPR model that was trained to generate estimates of the post-treatment FAS scores for each of the above-mentioned motor tasks. The estimated FAS scores were combined using a linear equation to compute the total (i.e., cumulative) FAS score, which was normalized by the maximum FAS score and reported in the following as a percentage score.

#### 1) Data Segmentation:

The sensor time-series recorded during the performance of the *Reaching Tasks* and the *Manipulation Tasks* were analyzed by first identifying the segments corresponding to distinct movement components. Because the *Reaching Tasks* consisted of a single movement component, no segmentation was necessary for this set of tasks. Instead, the data collected during the performance of the *Manipulation Tasks* was segmented into three components: 1) moving the hand from the lap to the table to reach for the object, 2) transporting or manipulating the object to accomplish the task (e.g., for “lift can” moving the soda can close to the mouth and then putting it back on the table), and 3) moving the hand back to the lap. These movement components were obtained automatically by instrumenting the objects utilized for different tasks (i.e., soda can, pencil, cards, and key) with a custom-designed resistive sensor using copper tapes that enabled the detection of changes in the resistance value when subjects touched or released the objects.

#### 2) Data Feature Extraction and Selection:

Data features that were previously shown to be relevant to capturing movement quality (i.e., speed, smoothness, and coordination) were extracted from the sensor data [30], [43]. First, the time-series for each axis of the accelerometer units, sampled at 51.2 Hz, were low-pass filtered (6th order Butterworth filter) with an 8 Hz cut-off frequency to attenuate high-frequency signal components not relevant to the proposed analyses. The velocity and displacement time-series for each axis of the sensor unit were derived by integrating the filtered accelerometer data using the trapezoid rule. A high-pass filter (6th order Butterworth filter) with a 0.25 Hz cut-off frequency was applied after integrating the time-series to attenuate the integration-drift component. The acceleration time-series were also differentiated to generate jerk time-series for each axis. Each of the three-dimensional displacement, velocity, acceleration, and jerk time-series were processed to derive the magnitude time-series defined as the square root of the components’ squares for each data sample.

Then, the following data features were extracted from each of the aforementioned time-series: 1) minimum, maximum, and mean values, 2) root mean square value, 3) ratio of the magnitude of the dominant frequency and total signal energy, 4) skewness, 5) kurtosis, 6) signal entropy, 7) Spearman correlation coefficients derived from the time-series computed for different axes, and 8) duration of the data segments associated with the performance of each movement component. The skewness and kurtosis represent the symmetricity and peakedness of the distribution of the time-series data, respectively. The entropy captures the regularity of the fluctuations observed in the time-series. The Spearman correlation coefficients of the acceleration magnitude time-series of all pair combinations of the sensor data (i.e., sensors positioned on the fingers, wrist, arm, and sternum) were computed. These correlation coefficients were meant to capture the characteristics of relative movements of different body segments, including compensatory movements such as leaning forward with the trunk when subjects reached for an object in front of them [30], [43]. For the *Reaching Tasks*, a total of 357 data features were extracted using data segments corresponding to the performance of the entire task. For the *Manipulation Tasks*, a total of 1,035 data features were extracted from each of the data segments corresponding to the performance of the above-mentioned movement components (see Data Segmentation).

A correlation-based data feature selection algorithm [44] was employed to identify a subset of data features suitable to capture the movement characteristics observed by clinicians during the performance of the FAS tasks. The algorithm selects data features that maximize the correlation with the dependent variable (i.e., the FAS score) and minimize the correlation among the data features (i.e., the degree of redundancy). This algorithm was deemed an appropriate choice because the dependent variable was numerical, and its value represented gradual, incremental changes in the quality of movements.

#### 3) Estimation of FAS:

The GPR algorithm was used to construct the machine learning model to estimate the FAS scores for each of the motor tasks shown in Fig. 3 using data features that were selected, as explained above. The GPR is a supervised, non-parametric learning method developed to estimate real-valued dependent variables using a collection of a finite number of continuous or nominal data features. GPR models have been previously applied to a large variety of machine learning problems of significant clinical relevance [45]–[47]. The work herein presented employed the squared exponential covariance function, and the hyperparameters were determined using a method that integrated *a posteriori* estimates to produce the conditional distribution of the latent variable, because this method performs well for training sets of small size [48]. A detailed overview of the GPR approach can be found in the book by Rasmussen and Williams [49].

The estimated FAS scores derived for each repetition of the motor tasks shown in Fig. 3 were first averaged per motor task. Then, the average scores of the eight motor tasks per patient visit were summed to derive a combined score: *FAS*_8 *Tasks*_. This score was used as input to a linear equation that was used in a prior study by our research group [30] (although not explicitly reported in the previous manuscript) and found to be suitable to estimate the total FAS score:

(1) $$FAS_{\text{total }}=\left(FAS_{8\;\text{Tasks}}\times 1.78\right)+2.97.$$

In the following, the *FAS*_*total*_ value is shown as percentage of the maximum attainable score (i.e., the 75 points of the scale when all 15 tasks are assigned a score equal to 5).

#### 4) Cross-Validation Technique and Evaluation Measures:

We used the Leave-One-Subject-Out Cross-Validation (LOSOCV) technique to evaluate the estimation performance of the GPR model. That is, we left out one subject’s post-treatment sensor data as the testing dataset while using the baseline and post-treatment sensor data belonging to the rest of the subjects to build the GPR model (i.e., training dataset). The trained GPR model was then applied to the left-out testing subject’s post-treatment sensor data to estimate the subject’s total FAS score at the post-treatment visit. This process was iterated until all subjects’ data was used as the testing dataset, and the overall estimation results were evaluated by comparing the estimated FAS scores and the actual FAS scores provided by the clinicians. The accuracy of the estimated FAS scores was evaluated by computing the Root Mean Square Error (RMSE), Pearson’s correlation coefficient *r*, bias, and standard deviation of the estimates.

### D. Clinical Data-Based Estimation

A schematic representation of the data analysis pipeline for the estimation of the post-treatment FAS scores based on clinical data is shown in Fig. 4 (block diagram on the right). Clinical variables utilized in the study were chosen based on previous work that investigated predictors of rehabilitation outcomes in chronic stroke survivors and TBI survivors among the variables that are readily available in the clinic [50], [51]. These variables were 1) the chronicity at baseline (i.e., the number of days from the acquired brain injury onset to the baseline), 2) the subject’s age at baseline (in years), 3) the length of the rehabilitation treatment (number of days between the baseline and post-treatment visits), 4) the number of rehabilitation therapy sessions between the baseline and post-treatment visits, 5) the FMA-UE score at baseline, and 6) the FAS score at baseline. These independent variables were used to estimate the difference in the FAS score between the baseline and the post-treatment visit. A GPR model was employed to obtain estimates of the change in the FAS score between the baseline and the post-treatment visit. The estimated value was then added to the baseline FAS score to derive the post-treatment FAS score. We employed the LOSOCV technique to evaluate the clinical data-based estimation model.

### E. Aggregating Sensor and Clinical Data-Based Estimates

This section describes the machine learning model that we developed to combine the FAS estimates derived using the sensor data (Section C) and the estimates derived using the clinical data (Section D) to improve the overall estimation performance (block diagram on the bottom of Fig. 4). The hypothesis motivating the development of this processing module is that clinical information related to the subject’s characteristics at baseline (e.g., the severity of motor impairments assessed using the FMA-UE scale) and the characteristics of the intervention (e.g., the number of rehabilitation sessions) are associated with the magnitude of the improvement observed in response to the rehabilitation intervention. Hence, a processing module can be used to optimally choose the weights to be assigned to the FAS estimate derived using the sensor data and the FAS estimate derived using the clinical data, thus resulting in a more accurate estimate of the post-treatment FAS score. More specifically, the clinical variables were analyzed to derive a linear equation that assigns weights to the sensor data-based estimates and the clinical data-based estimates to maximize the estimation accuracy of the post-treatment FAS score. This approach is appealing because it allows one to establish a model generating post-treatment FAS score estimates that accounts for differences in the characteristics of the subjects as well as of the rehabilitation interventions implemented in different individuals.

The proposed method employed a linear model that combined the two FAS estimates using the following equation:

$$\hat{y}=\omega_{c}\;{\hat{y}}_{c}+\omega_{s}\;{\hat{y}}_{s},$$

where ${\hat{y}}_{s}$ and ${\hat{y}}_{c}$ denote the total FAS score estimates based on the sensor and clinical data, respectively. In addition, *ω*_*s*_ and *ω*_*c*_ are the model parameters and obey the equation *ω*_*s*_ + *ω*_*c*_ = 1. Hence the equation above can be rewritten as follows:

(2) $$\hat{y}=\omega_{c}{\hat{y}}_{c}+\left(1-\omega_{c}\right){\hat{y}}_{s}$$

where 0 ≤ *ω*_*c*_ ≤ 1.

The value of *ω*_*c*_ was determined using a training set D obtained from *n* subjects: $D=\left(X_{c},{\hat{y}}_{c},{\hat{y}}_{s},y\right)=\left\{\left(x_{c,i},{\hat{y}}_{c,i},{\hat{y}}_{s,i},y_{i}\right)|i=1,\cdots n\right\}$ where ***X***_*c*_ is an input *n* × *m* matrix of normalized clinical variables with zero-means and unit-variances, *m* denotes the number of clinical variables (i.e., *m* = 6), and ***y*** is a *n* × 1 vector of the target (dependent) variable (i.e., post-treatment total FAS score as determined by the clinicians). The model parameters were computed as a linear function of the clinical variables (i.e., *ω*_*c*,*i*_ = ***x***_*c*,*i*_ ***θ***) for all *i*, which yielded the following equation:

$$\hat{y}=X_{c}\theta \circ {\hat{y}}_{c}+\left(1-X_{c}\theta \right)\circ {\hat{y}}_{s}\;=X_{c}\theta \circ {\hat{y}}_{c}+{\hat{y}}_{s}-X_{c}\theta \circ {\hat{y}}_{s}\;=X_{c}\theta \circ \left({\hat{y}}_{c}-{\hat{y}}_{s}\right)+{\hat{y}}_{s}$$

subject to 0 ≤ ***X***_*c*_***θ*** ≤ 1. ***θ*** is a *m* × 1 vector of model parameters. The notation ○ denotes the Hadamard (element-wise) product of two vectors. The training algorithm was designed to find the value of ***θ*** that minimizes the error between $\hat{y}$ and ***y***. To resolve this problem, we defined the following cost function:

$$J(\theta )={\left(y-\hat{y}\right)}^{T}(y-\hat{y})\;={\left[y-X_{c}\theta \circ \left({\hat{y}}_{c}-{\hat{y}}_{s}\right)-{\hat{y}}_{s}\right]}^{T}\;\times \left[y-X_{c}\theta \circ \left({\hat{y}}_{c}-{\hat{y}}_{s}\right)-{\hat{y}}_{s}\right]\;={\left[\left(y-{\hat{y}}_{s}\right)-X_{c}\circ \left({\hat{y}}_{c}-{\hat{y}}_{s}\right)\theta \right]}^{T}\;\times \left[\left(y-{\hat{y}}_{s}\right)-X_{c}\circ \left({\hat{y}}_{c}-{\hat{y}}_{s}\right)\theta \right]$$

Then, by introducing a matrix $A=X_{c}\circ \left({\hat{y}}_{c}-{\hat{y}}_{s}\right)$ and a vector $b=\left(y-{\hat{y}}_{s}\right)$, we re-wrote the above-defined cost function as follows:

$$J(\theta )={\left(b-A\theta \right)}^{T}(b-A\theta )\;=\theta^{T}A^{T}A\theta -2b^{T}A\theta$$

Accordingly, the optimal parameter θ* was defined as

$$\theta^{*}=\underset{\theta }{\text{argmin}}J(\theta ),$$

subject to 0 ≤ ***X***_*c*_***θ*** ≤ 1. The cost function *J*(***θ***) is a quadratic function of ***θ***, and thus we employed quadratic programming to find the optimal solution θ* subject to 0 ≤ ***X***_*c*_θ* ≤ 1. Similarly, we employed the LOSOCV technique to evaluate the proposed constrained linear model. The total FAS score of the test dataset was obtained by applying the optimized model parameter θ* to (2) using $\omega_{c}=x_{c}^{\prime }\theta^{*}$, where $x_{c}^{\prime }$ denotes the clinical information of the subject whose data is used to define the test dataset.

Two simple benchmark methodologies were considered to compare the estimation performance of the proposed method. The first benchmark was derived by averaging the sensor data-based estimates and the clinical data-based estimates with equal weights, i.e., *ω*_*c*_ = 0.5 in (2). Herein, we refer to this method as the *averaging method*. The second benchmark was derived by computing the weights based on the normalized variance (as a measure of uncertainty) of the two estimates $\omega_{c}=V\left[{\hat{y}}_{c}\right]/\left(V\left[{\hat{y}}_{c}\right]+V\left[{\hat{y}}_{s}\right]\right)$ and $\omega_{s}=V\left[{\hat{y}}_{s}\right]/\left(V\left[{\hat{y}}_{c}\right]+V\left[{\hat{y}}_{s}\right]\right)$, where $V\left[{\hat{y}}_{c}\right]$ and $V\left[{\hat{y}}_{s}\right]$ represent the predictive variance (a measure of uncertainty) of the estimates derived using the GPR models based solely on the clinical data and solely on the sensor data, respectively. Herein, we referred to this method as the *variance-based method*.

An additional benchmark was derived by selecting the better one between the two estimates obtained using solely the sensor data and solely the clinical data. The corresponding accuracy value is herein referred to as the *upper bound*. This approach is of no practical interest, because one cannot establish *a priori* which estimate is more accurate (i.e., the one derived by using solely the sensor data or the one derived using solely the clinical data). However, this approach provides a suitable benchmark because it is the best possible combination of the above-mentioned estimates if they are biased in the same direction (i.e., they both have a positive bias or they both have a negative bias).

## III. Results

Table I shows 1) the baseline total FAS scores provided by the clinicians, 2) the post-treatment FAS scores, and 3) the changes in FAS score in response to the rehabilitation intervention (i.e., post-treatment vs. baseline score) for stroke and TBI survivors who participated in the study. All FAS scores were computed according to (1). A comparison between the stroke vs. TBI survivors’ data did not show any statistical differences for the baseline scores (*t*-test for unpaired samples, *p* ≅ 0.40), post-treatment scores (*p* ≅ 0.19), and the changes in FAS score (*p* ≅ 0.39). These results indicate that baseline and post-treatment movement quality (as captured by the FAS scores) and the change in movement quality in response to the rehabilitation intervention displayed similar characteristics in the study samples of stroke and TBI survivors. Hence, the sensor and clinical datasets obtained from the two populations were combined in a single dataset – with a total number of subjects equal to 44 at baseline and 38 post-treatment – to build the above-described models and derive estimates of the FAS scores for each study participant.

**Fig. 5.**
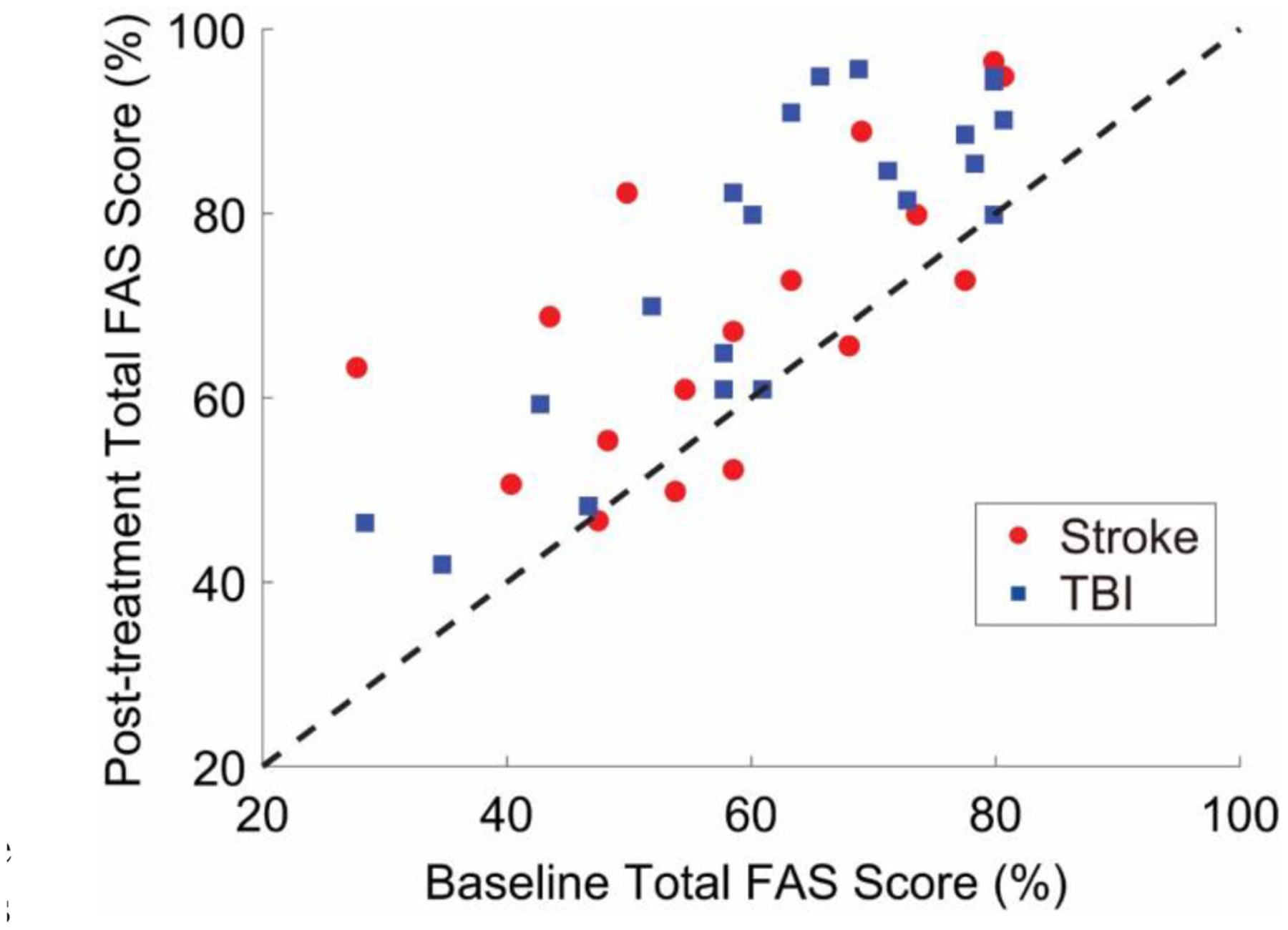
Post-treatment FAS scores vs. baseline FAS scores. FAS scores herein represented were generated by clinicians via observation of the motor patterns of stroke and TBI survivors. The data shows a large variability in the response to the rehabilitation intervention across study participants. The 45-degree line is shown as reference (i.e., data on the 45-degree line are marked by equal post-treatment and baseline scores).

**Fig. 6.**
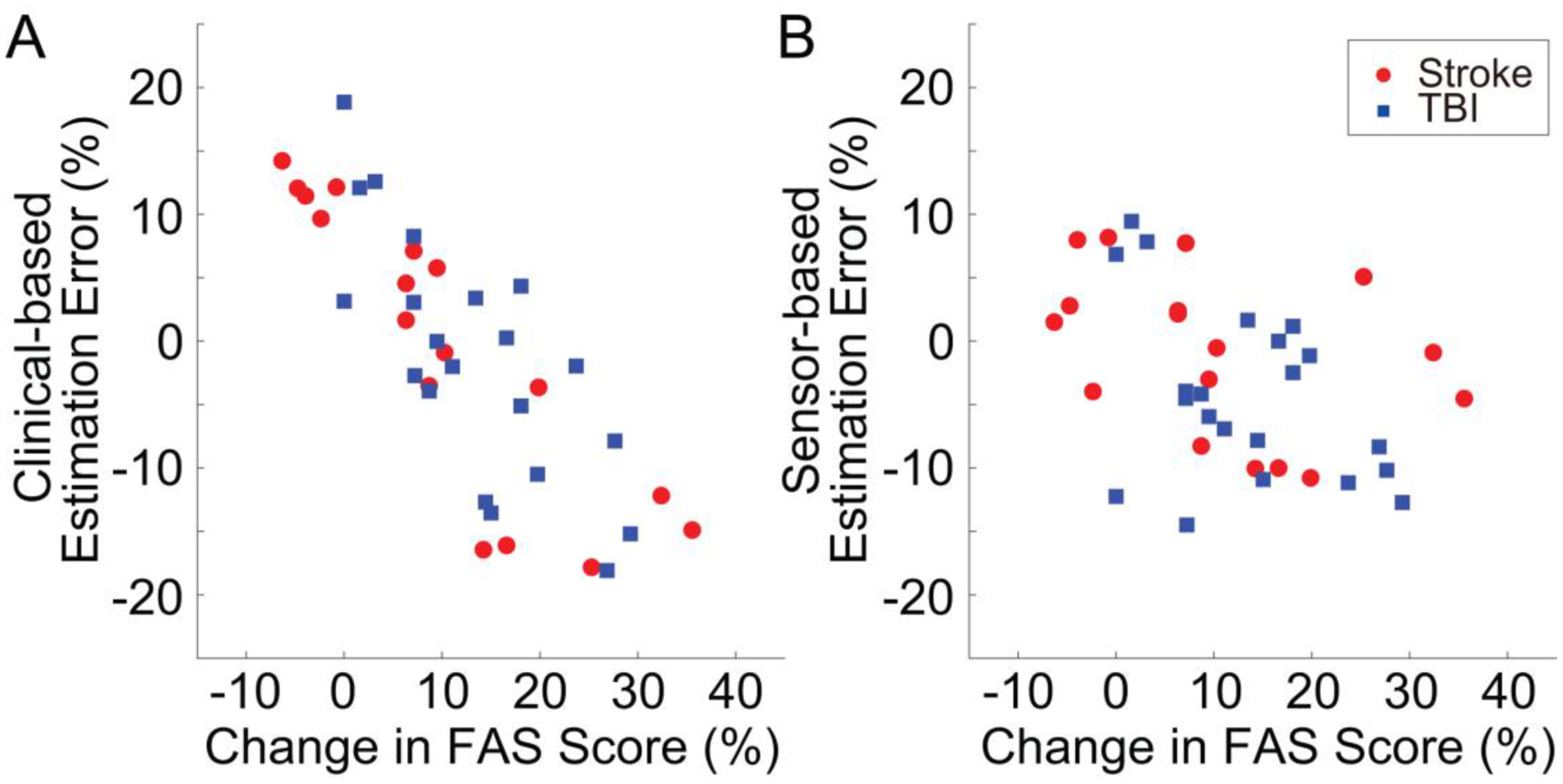
Estimation error vs. change in FAS score (i.e., post-treatment FAS score - baseline FAS score) for estimates derived using solely the clinical data (panel A) and using solely the sensor data (panel B). A distinct pattern is observed in the estimation error for the clinical data-based estimates, thus indicating that the algorithm overestimated the change in FAS score for subjects who displayed a minimal response to the intervention and underestimated it for subjects who displayed a large response to the intervention. In contrast, sensor data-based estimates appear to be randomly distributed across the range of change in FAS score that marked the response to the rehabilitation intervention of subjects enrolled in the study.

Fig. 5 shows the post-treatment vs. the baseline total FAS score data for all the study participants. Although stroke and TBI survivors on average demonstrated similar improvements in movement quality in response to the rehabilitation intervention, the responses at the individual level were highly variable and did not show any notable pattern. This was – to some extent – captured by both estimation algorithms using solely the sensor data and solely the clinical data. Table II shows the estimation errors that marked the two estimation procedures. The sensor data-based estimation procedure resulted in a RMSE of 7.7% with a Pearson’s correlation of 0.91 when compared to the actual FAS scores. The clinical data-based estimation procedure resulted in a RMSE of 10.8% with a Pearson’s correlation of 0.79. The smaller correlation value obtained for the clinical data-based estimates appears to be – at least in part – due to the different bias characteristics of the two estimation algorithms, as shown in Fig. 6. The plot on the left shows the estimation error vs. the actual change in FAS score (i.e., post-treatment vs. baseline score) for the clinical data-based estimation algorithm, whereas the plot on the right shows the same data for the sensor data-based estimation algorithm. It is observed that the clinical data-based estimation algorithm is marked by a distinct relationship between the estimation error and the actual change in FAS score. The plot shows that the algorithm generally overestimated the change in FAS score for subjects whose FAS score improved minimally (or degraded) in response to the rehabilitation intervention, whereas it generally underestimated the change in FAS score for subjects whose FAS score improved considerably in response to the intervention. In other words, the clinical data-based estimation algorithm led to relatively accurate estimates for those subjects whose FAS score improved in an amount similar to the average of the population, but failed to generate accurate estimates when subjects displayed a response to the rehabilitation intervention that deviated from the average response. On the other hand, the sensor data-based approach did not show any obvious trends in the estimation error, which appears to underlie the superior estimation performance compared to the clinical data-based estimation shown in Table II.

Table II also shows the results obtained using the *averaging method*, the *variance-based method*, and the *upper-bound method*. As anticipated, the *upper-bound method* outperformed all the methods tested in the study and showed better results than all the other benchmarks. The *upper-bound method* led to a RMSE of 6.0% and a Pearson’s correlation of the derived estimates with the actual FAS scores of 0.94. The proposed method combined in a very effective way the sensor data-based estimates and the clinical data-based estimates. Using the proposed method led to a RMSE of 6.9% with estimates marked by a Pearson’s correlation with the actual FAS scores of 0.94 (see Table II). The bias and the standard deviation of the estimates were −2.5% and 6.8%, respectively. These results compare favorably to the inter-rater reliability data for the FAS [38]. It is worth noticing that the algorithm based solely on the sensor data did not always perform better than the algorithm based solely on the clinical data. The sensor data-based estimates were more accurate 68.4% of the time. The clinical data-based estimates were more accurate 31.6% of the time.

**Fig. 7.**
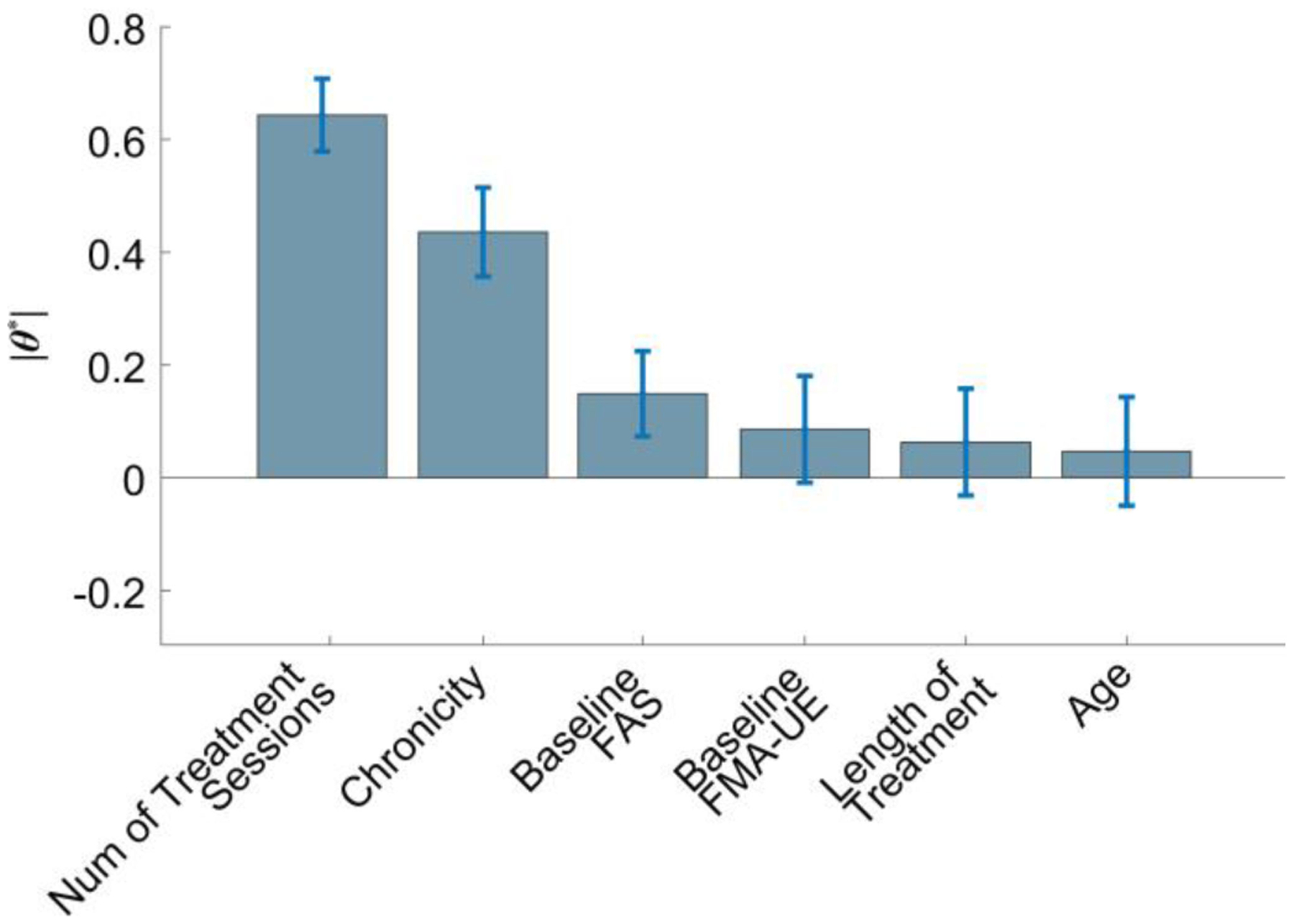
Average and standard deviation of the values of θ* that were computed from the iterations of the leave-one-subject-out cross-validation of the proposed algorithm to combine clinical data-based and sensor data-based estimates. The values represent the contributions of the examined clinical parameters to derive the weights to be used to combine the sensor data-based and clinical data-based estimates. The estimates were combined using the aggregation module shown in Fig. 1.

The improvement in the accuracy of the FAS estimates obtained using the proposed algorithm to combine sensor and clinical data-based estimates compared to the estimates derived using solely the sensor data was the result of deriving appropriate θ* values to determine an optimal *ω*_*c*_ value, according to (2). Insights into the level of contributions (or importance) of different clinical variables in determining the value of *ω*_*c*_ can be derived by examining the average and standard deviation of the θ* values computed over the iterations of the LOSOCV shown in Fig. 7. The number of treatment sessions, chronicity, and baseline FMA-UE value contributed with positive θ* values to determine the optimal value of *ω*_*c*_. The θ* values were equal to 0.61 ± 0.06, 0.41 ± 0.07, and 0.08 ± 0.09, respectively. The baseline FAS value was associated with a negative θ* value equal to −0.14 ± 0.07. The age of the subjects and length of treatment had a relatively modest impact on determining the optimal value of *ω*_*c*_. Their θ* values were equal to 0.04 ± 0.09 and −0.06 ± 0.09, respectively.

It should be emphasized that the mean and standard deviation of the weights (*ω*_*c*_ and *ω*_*s*_ = 1 − *ω*_*c*_) in (2) computed over the LOSOCV iterations showed that the proposed algorithm generally assigned a larger weight to the sensor data-based estimates. On average, 7% of the weight was allocated to *ω*_*c*_ and 93% to *ω*_*s*_. For the subset of data points for which the clinical data provided more accurate estimates, 13% of the weight was allocated to *ω*_*c*_ and 87% to *ω*_*s*_. On the other hand, for the subset of data points for which the sensor data provided more accurate estimates, only 4% of the weight was allocated to *ω*_*c*_ and 96% to *ω*_*s*_.

**Fig. 8.**
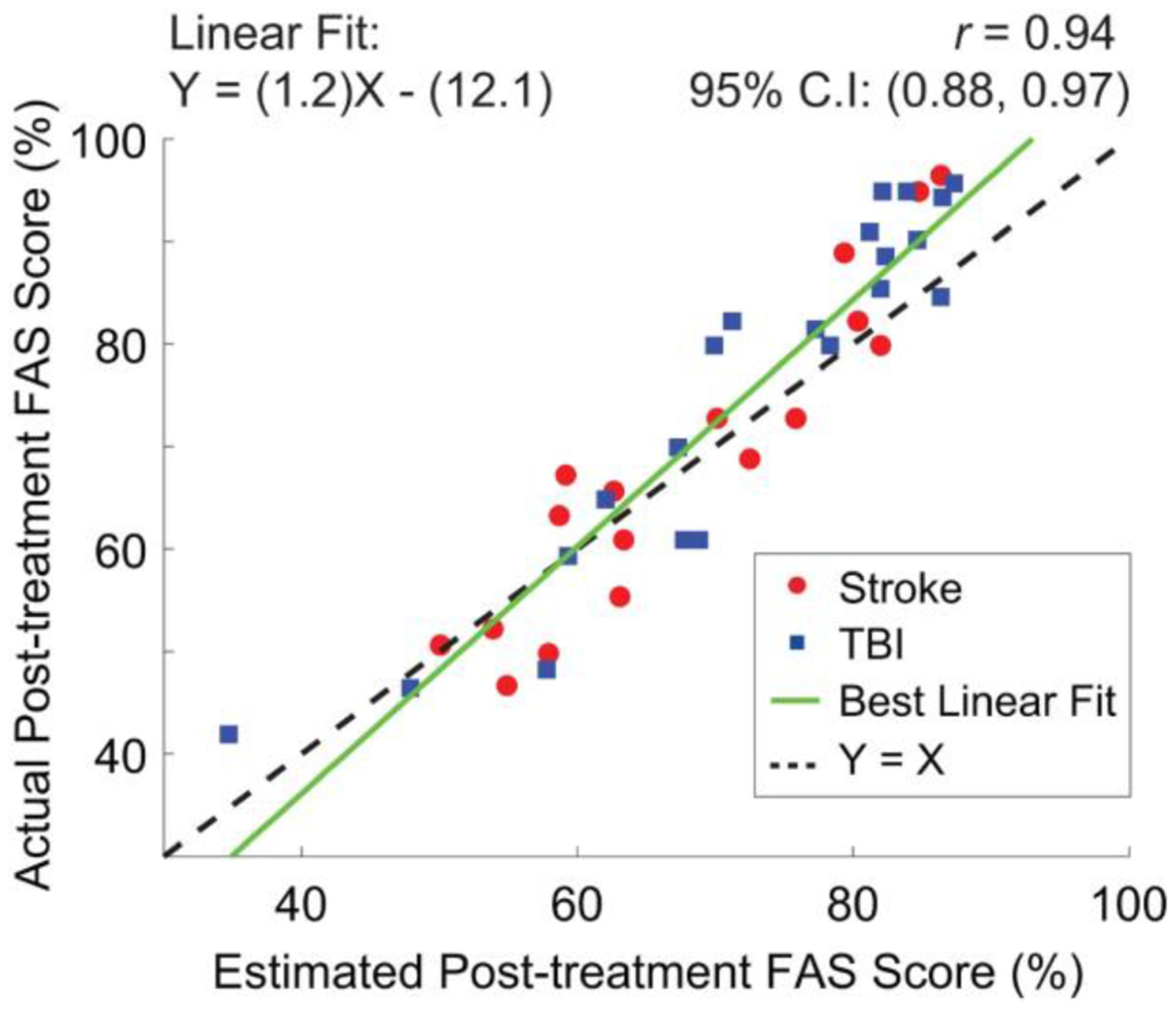
Scatter plot of the post-treatment FAS scores generated by clinicians via observation of movement patterns in stroke and TBI survivors vs. estimates derived using the proposed algorithm to combine sensor data-based and clinical data-based estimates.

The total FAS scores derived using the proposed algorithm closely matched the actual FAS scores provided by the clinicians, as shown in Fig. 8. The results were marked by a statistically significant correlation (Pearson’s correlation, *p* < 0.01). Interestingly, when combining the results for stroke and TBI survivors, the estimation error was equal to 6.1 ± 3.8%. The results for the stroke survivors were marked by an estimation error equal to 5.43 ± 3.53%, whereas the results for the TBI survivors were marked by an estimation error equal to 6.6 ± 3.92%. No statistically significant difference was observed between the two groups (*t*-test for unpaired samples, *p* ≅ 0.17).

## IV. Discussion

Large variability in the response of stroke and TBI survivors to rehabilitation interventions marked the results of this study (Fig. 5). Prior work investigated the relationship between rehabilitation outcomes and several clinical variables, including the intervention dosage (i.e., number of treatment sessions or hours of therapy) [7], [10], [11], the chronicity level (i.e., time since acquired brain injury) [5]–[8], and the outcome of baseline clinical assessments [7], [8]. Age (because of the decrease in neural plasticity with aging [52]) and length of treatment (because of the proportional recovery observed in the early phases post-injury [53]) have also been considered as potential predictive factors of rehabilitation outcomes.

The GPR model utilized in this study to generate FAS score estimates from baseline clinical data performed better than traditional regression models utilized in previous work to predict FMA-UE scores in stroke survivors [5], [7] and Functional Independence Measure scores in TBI survivors [8]. While it remains to be seen if this is a specific characteristic of the FAS, the analysis of the estimation error presented in Fig. 6 showed that the algorithm that we developed to estimate the FAS scores using solely clinical data failed to accurately capture the variability across subjects that we observed in response to rehabilitation interventions. In contrast, the estimation algorithm based solely on the sensor data generated equally accurate estimates of the FAS scores for subjects who responded well or minimally to the rehabilitation intervention. This result shows a significant advantage of using wearable sensors to monitor rehabilitation outcomes. Nonetheless, our results showed that the accuracy of sensor data-based estimates can be improved by combining them with clinical data-based estimates using weights chosen on the basis of the subject’s clinical characteristics at baseline and the characteristics of the intervention (Table II). Importantly, the estimation error that marked the FAS predictions obtained using the proposed method is of a magnitude similar to the variability across raters that one would expect based on available data [38].

Key variables to determine how to combine clinical data-based estimates and sensor-data based estimates were shown to be the number of treatment sessions, the chronicity of the neurological injury, and the FAS and FMA-UE scores at baseline. This result is of great interest because it is consistent with previous reports indicating that large motor gains can be achieved with high-dosage interventions [7], [11] but it is in conflict with other reports suggesting that an increase in therapy dosage does not necessarily lead to improvements in motor abilities [10]. It is also consistent with previous observations of the relationship between motor gains and chronicity [5]–[8] as well as of the relationship between rehabilitation outcomes and FMA-UE scores in stroke survivors [5], [7] and Functional Independence Measure scores in TBI survivors [8] collected at baseline.

Whereas the objective of this study was to develop a framework to generate FAS score estimates by combining clinical data-based estimates and sensor data-based estimates, future work should be devoted to leveraging the proposed framework to generate more accurate estimates of rehabilitation outcomes by improving both the algorithm to derive estimates using solely sensor data and the predictions derived using solely clinical data. Because, in the context of a clinical application of the proposed approach, sensor data would be collected longitudinally, then techniques to model the time-course of the FAS scores could be utilized to improve the accuracy of sensor data-based estimates. These include GPR models suitable to predict datapoints of a time-series [49]. In addition, to improve the accuracy of FAS estimates generated using clinical data, it is appealing to consider going beyond data that is routinely available in the clinic. Previous studies have shown the relevance of measures of corticospinal tract integrity to predict motor gains in the early phases of the recovery process in both stroke [54]–[56] and TBI [57] survivors. Although imaging data to assess corticospinal tract integrity is not typically available in medical records, its relevance to predict clinical outcomes in stroke and TBI survivors provides strong motivation to collect and integrate such information in future studies. Furthermore, recent results have highlighted that measures of the integrity of the somatosensory system at baseline account for a significant portion of the variability in response to rehabilitation of stroke survivors [58]. These measures should be merged with metrics aimed to genotyping [14], [15] and motor-phenotyping [21], [22] individuals at baseline as means to improve the accuracy of the prediction of rehabilitation outcomes. These measures might improve the accuracy of algorithms combining clinical data-based estimates and sensor data-based estimates.

It is worth emphasizing that the results presented in this manuscript support a clinical scenario in which stroke and TBI survivors’ recovery trajectories are longitudinally monitored to develop patient-specific rehabilitation interventions. Clinical data collected at baseline could be utilized to compute the expected clinical outcomes of the intervention (assuming responsiveness to the prescribed treatment). Sensor data collected longitudinally in the clinic or in the home and community settings during the performance of functional motor tasks (such as those shown in Fig. 3) could be analyzed to monitor progression. Sensor-based estimates would allow clinicians to evaluate patients’ response to the prescribed treatment and provide the opportunity to adjust the intervention and hence achieve optimal clinical outcomes. For example, a significant difference between the results of the clinical data-based vs. sensor data-based estimates would suggest that the patient’s recovery trajectory is deviating from the recovery trajectory that is expected based on his/her clinical characteristics. This observation would trigger an adjustment in the therapeutic intervention. We look upon this approach as key to implement the next generation of precision rehabilitation interventions, namely the implementation of individually tailored rehabilitation interventions.

A few limitations of the work herein presented should be acknowledged. First, the study involved a limited number of study participants. We trained our machine learning models based on sensor and clinical data collected from a total of 44 subjects (22 stroke and 22 TBI survivors) and evaluated the trained models based on data collected during the post-treatment visits from a total of 38 subjects (17 stroke and 21 TBI survivors). However, despite the relatively small data samples, the evaluation of the machine learning models was performed using the LOSOCV technique, which minimized the chances of overfitting the training model to patient-specific movement patterns. Secondly, the proposed approach is based on collecting sensor data longitudinally, during the intervention period. Sensor data could be collected in the clinic as part of the outpatient visits scheduled for treatment or in the home and community settings. In the former case, therapists would assist patients in donning/doffing the sensors. In the latter case, either patients would have adequate motor and cognitive abilities to don/doff the sensors on their own or a caregiver would provide assistance. In all these scenarios, minimizing the number of sensors would be highly desirable, which we did not pursue in this study. Future work should identify the minimum set of wearable sensors and optimal body locations that can provide adequate tracking of the recovery trajectory [35]. Lastly, the size and shape of the wearable sensors that we used to collect accelerometer data was adequate for the study but would not be ideal for long-term monitoring. Future studies should consider using Band-Aid like units for placement on the sternum and arm [59] and ring-sensors to monitor movements of the fingers [28].

## V. Conclusion

The study showed that a GPR model based on clinical data collected at baseline can predict rehabilitation outcomes (i.e., FAS scores) in stroke and TBI survivors better than traditional models, but still fails to account for the variability in the level of responsiveness to treatment across individuals. A GPR model was also used to estimate rehabilitation outcomes based on data collected using wearable sensors with results superior to those obtained using clinical data. Using constrained linear modeling and quadratic programming, we developed an algorithm to combine the clinical data-based estimates and the sensor data-based estimates. The algorithm accounted for the clinical characteristics of each subject at baseline. The FAS score estimates derived using the proposed algorithm showed a high level of correlation with FAS scores provided by clinicians (*r* = 0.94). These estimates were marked by an error (RMSE = 6.9%, bias = −2.5%, standard deviation = 6.8%) that compared favorably with available inter-rater reliability data for the FAS. The algorithms developed in this study can be used to predict and monitor rehabilitation outcomes and enable the development of patient-specific rehabilitation interventions based on the responsiveness of each individual to different treatment strategies.

## Figures and Tables

**TABLE I:** A Summary (mean ± std. dev.) of the Baseline, Post-Treatment, and Change in the FAS Scores in Stroke and TBI Survivors who Participated in this Study

	Stroke	TBI
Baseline FAS Score	58.5 ± 15.1%	62.7 ± 15.3%
Post-Treatment FAS Score	68.7 ± 15.6%	76.0 ± 17.3%
Change in FAS Score	14.2 ± 12.6%	17.2 ± 9.0%

**TABLE II:** A Summary of Estimation Performance of the Proposed Method and a Number of Comparative Benchmark Techniques

	RMSE	*r* (95% C.I)	Bias	Standard Deviation
Sensor data-based Estimation	7.7%	0.91 (0.85, 0.96)	−2.9%	7.2%
Clinical data-based Estimation	10.8%	0.79 (0.63, 0.88)	−0.96%	10.9%
Equal Weighting (Averaging)	8.3%	0.90 (0.81,0.95)	−1.9%	8.2%
Variance-based Weighting	7.5%	0.92 (0.85, 0.96)	−2.6%	7.2%
Upper Bound	6.0%	0.94 (0.90, 0.97)	−1.0%	6.0%
**Proposed Method**	**6.9%**	**0.94 (0.88, 0.97)**	**−2.5%**	**6.8%**

RMSE stands for root mean square error and *r* represents the Pearson’s correlation coefficient.
